# EMS Stretcher “Misadventures” in a Large, Urban EMS System: A Descriptive Analysis of Contributing Factors and Resultant Injuries

**DOI:** 10.1155/2012/745706

**Published:** 2012-04-23

**Authors:** Jeffrey M. Goodloe, Christopher J. Crowder, Annette O. Arthur, Stephen H. Thomas

**Affiliations:** ^1^Department of Emergency Medicine, University of Oklahoma School of Community Medicine, Tulsa, OK, USA; ^2^College of Medicine, The University of Oklahoma Health Sciences Center, Oklahoma City, OK, USA

## Abstract

*Purpose*. There is a paucity of data regarding EMS stretcher-operation-related injuries. This study describes and analyzes characteristics associated with undesirable stretcher operations, with or without resultant injury in a large, urban EMS agency. *Methods*. In the study agency, all stretcher-related “misadventures” are required to be documented, regardless of whether injury results. All stretcher-related reports between July 1, 2009 and June 30, 2010 were queried in retrospective analysis, avoiding Hawthorne effect in stretcher operations. *Results*. During the year studied, 129,110 patients were transported. 23 stretcher incidents were reported (0.16 per 1,000 transports). No patient injury occurred. Four EMS providers sustained minor injuries. Among contributing aspects, the most common involved operations surrounding the stretcher-ambulance safety latch, 14/23 (60.9%). From a personnel injury prevention perspective, there exists a significant relationship between combative patients and crew injury related to stretcher operation, Fisher's exact test 0.048. *Conclusions*. In this large, urban EMS system, the incidence of injury related to stretcher operations in the one-year study period is markedly low, with few personnel injuries and no patient injuries incurred. Safety for EMS personnel and patients could be advanced by educational initiatives that highlight specific events and conditions contributing to stretcher-related adverse events.

## 1. Introduction

The majority of patient transportation in the prehospital emergency medical care environment involves the use of mobile stretchers. Stretcher utilization occurs in three distinct phases: (1) unloading from the ambulance; (2) loading into the ambulance; (3) transporting over surface structures. Several commercially manufactured devices have been designed to best accomplish these activities. Constraints on the stretcher system are myriad, including weight and size of the patient, ease of use, and durability. With these limitations in mind, finding the balance of performance and safety is an important mission.

 Thus, ambulance stretcher operations constitute a necessary, yet risky, part of the provision of prehospital emergency medical services. Despite generally widespread acknowledgement of risk to patient and EMS professional alike, there remains a paucity of data addressing ambulance stretcher associated injury analysis. A review of the past decade's literature in the PubMed database (http://www.ncbi.nlm.nih.gov/pubmed) searching with the unrestricted term of  “ambulance stretcher” yields few relevant investigations [1–5]. With the noted exception of Wang et al., these studies are largely simple ergonomic evaluations in nature. Wang et al. used the United States Food and Drug Administration's Manufacturer and User Facility Device Experience database to describe adverse events related to the ambulance stretcher. Their dataset captures significant events over a large time period. However, due to lack of a “denominator” (i.e., total number of transports over which stretcher mishaps were analyzed), they are unable to define an event rate. It is also likely that many stretcher events—perhaps some significant—went unreported to the USFDA database.

 While helpful in reminding EMS professionals that physical stressors are attendant to stretcher operations, the lessons of nearly all extant literature can best be summed as being primarily risk assumptive, without analysis of actual injuries sustained. Wang et al. provides the lone peer-reviewed study (to our knowledge) of any data measuring the injuries related to stretcher “adverse events.”

 As many in EMS will know, there is widespread recognition of the potential for EMS professionals to sustain back injury in the performance of basic patient transportation. Many entrepreneurial ventures utilize this concern of back injury risk in marketing new stretcher designs and stretcher lifting and loading capabilities. Certainly, no national organization tracks all relevant incidents of ambulance stretcher-related injuries. In the present era, despite the presence of many groups advocating for increased safety in prehospital emergency medical care, it appears unlikely there will be significant movement towards comprehensive reporting that could facilitate widespread evidence-based progress in limiting stretcher-related injuries.

 Against this important background, we sought to critically analyze the types and frequencies of injuries sustained by patients and EMS professionals directly related to stretcher operations occurring in a large, urban EMS system in the southwestern United States. This investigation was additionally conceived to design educational initiatives for purposes of injury prevention as well as to add knowledge in this important, yet underreported, arena.

## 2. Methods

### 2.1. Study Design

This was a retrospective, observational study of all patient transports conducted in a single large, urban EMS system. The event of interest was an injury sustained by a patient and/or EMS professional directly related to use and operation of the stretcher.

### 2.2. Study Setting and Population

The study EMS agency, Emergency Medical Services Authority (EMSA), is a public utility model agency serving central and metropolitan Oklahoma City and Tulsa, OK. The two geographical service areas combined comprise approximately 1100 square miles and are populated by 1,201,232 residents. [6]. In this study year, EMSA's ambulance fleet was comprised of 93 Type I modular units on the Ford F450 chassis. Ambulance design met federal emergency vehicle specifications [7]. The nonpowered, manual-lift stretchers were uniform, manufactured by Stryker Corporation, Portage, MI. There were no changes in stretcher design or operation during the study year. All EMS personnel operating the stretcher had received formal one-hour stretcher operation instruction, designed to thoroughly orient each personnel to the loading, movement, and unloading mechanisms of the stretcher. One stretcher type and model is exclusively utilized throughout the study EMS agency. Immediately after-instruction, all personnel exhibited desirable stretcher operation ability during 20–30 minutes of practical assessments involving loading, movement, and unloading exercises supervised by training officers. Personnel must complete the didactic and practical components described prior to unsupervised duty. An annual continuing education forum regarding patient safety is conducted, including brief discussion of patient movement responsibilities, though no formal ongoing education is scheduled to replicate the initial stretcher-related education. All field personnel at EMSA are certified at a minimum at the EMT level. Ambulance tours are 12 hours in duration. Ambulances are located throughout the service area, utilizing system status management designated posts.

### 2.3. Study Protocol

EMSA mandates that all adverse events related to stretcher transport of a patient, however minor in force or resultant injury, are reported by the involved EMS personnel utilizing incident reporting; these reports, with details entered as free text, are logged and maintained in a computerized database (Ninth Brain Suite, NinthBrain, Grand Rapids, MI). The study period was chosen by convenience and by inclusion of all weather seasons to be from July 1, 2009 to June 30, 2010.

All stretcher-related incident reports related to patient movements during this study year were queried. These incident reports were compiled by the Safety and Risk Manager for EMSA and reviewed in detail by two of the researchers (CJC, JMG) for appropriate inclusion in the study as well as for agreement upon descriptive characteristics of contributing factors and sustained injuries.

 Data recorded included the nature of the adverse event as well as any documented contributing factors, patient body habitus if notably obese, patient behavior if notably combative or otherwise physically disruptive, timing of incident (e.g., loading), and presence of suspected injury to the patient and/or EMS personnel. All events were categorized into three time periods: unloading, loading, and surface movement. Subtype characterizations included equipment failure, safety latch malfunction, poor surface conditions, obese patient, and uncooperative patient. The data were abstracted into a spreadsheet using Microsoft Excel (Microsoft Corporation, Redmond, WA).

### 2.4. Outcome Measures

The primary outcome measure was the rates of stretcher operation associated injury to patients and to EMS professionals. These rates were assessed using the analytical methods described below. The secondary outcome measure was assessment of the particular contributing factors and timeframes of stretcher operation associated with stretcher-related incident reports filed by EMS personnel. *A priori* analytical emphasis was placed upon unloading or loading patients. These timeframes are generally accepted as events of requiring higher energy to safely move patients, and thus, events that seemed particularly likely to contribute to injury.

### 2.5. Analytical Methods

The primary analytic approach followed the general lines of the reporting in the previous study by Wang et al. Because of our having the total number of “exposures” (transports), we were also able to execute event rate calculations.

 The number of stretcher events was determined, and an event rate per 1,000 transports was calculated; 95% Poisson confidence intervals (CIs) were then calculated around the overall estimate for event rates. For construction of CIs in which the point estimate was zero (e.g., patient injuries), a one-sided 97.5% CI was calculated. For proportional data (e.g., percentages of stretcher events with crew injury), binomial exact 95% CIs were calculated.

 For comparative analyses, lack of overlap between CIs was taken as indicative of statistical significance. For tabular data, results were analyzed using Fisher's exact text. For analyses, significance was defined at the *P* < 0.05 (95% CI) level. STATA 11 MP (StataCorp, College Station, TX) was used for all testing.

### 2.6. Institutional Review Board Review

This study protocol was reviewed and approved by The University of Oklahoma Health Science Center Institutional Review Board.

### 2.7. Study Association and Funding

The entire costs of this study were borne by EMSA and the OU Department of Emergency Medicine. Stryker Corporation (the manufacturer of the stretchers utilized throughout the study period) had no association with (or knowledge of) this study. Stryker Corporation did not fund the study and did not provide study-associated product pricing to EMSA.

## 3. Results

During the study year period, the EMS agency transported 129,110 patients. There were 23 adverse stretcher events reported, yielding an event rate of 0.018 per 1,000 transports. Stretcher events were relatively evenly divided (44% and 56%) between the service's two geographic locations (Oklahoma City and Tulsa). There were no patient injuries (1-side 97.5% CI 0 to 0.28 per 1,000 transports). There were 4 EMS provider injuries, event rate 0.031 per 1,000 transports (95% CI 0.01 to 0.08 per 1,000 transports). All EMS injuries were minor and no time off was requested or required due to these injuries. These included 2 knee injuries and 2 back injuries.

 The timing of reported adverse stretcher events was analyzed (Table 1). The majority occurred during unloading 15/23 (65.2%; 95% CI 42.7–83.6%). 5 of 23 (21.7%; 95% CI 7.5–43.7%) events occurred during loading and 3 of 23 (13.0%; 95% CI 2.8%–33.6%) events occurred during surface movement.

In addition to the timing of the event, we also investigated contributing factors (Table 2). In some cases there were multiple factors. The most common cause of an adverse event was a stretcher-ambulance safety latch malfunction 14/23 cases (60.9%; 95% CI 38.5%–80.3%). Poor surface conditions contributed to 4/23 cases (17.4%; 95% CI 5.0%–38.8%). In the four surface conditions reviewed each contributing to a stretcher/patient drop, two events were caused by wheels of the stretcher becoming caught in an uneven surface, specifically a crack in a paved road and on a gravel parking lot. The remaining two poor surface conditions were described as ice-covered sloping driveways contributing to both slippery movement and patient imbalance on the stretcher mattress. Equipment failure occurred in 3/23 cases (13.0%; 95% CI 2.8%–33.6%). In these three equipment failures reviewed, each contributing to a stretcher/patient drop, two events were caused by stretcher undercarriage failure. In one case, the undercarriage failed to lower by manual lever engagement during unloading. In the other undercarriage failure, the stretcher legs became stuck during a loading attempt. The remaining instance of equipment failure was described as the ambulance floor-mounted safety latch breaking while the stretcher was being unloaded. In 2/23 cases (8.7%; 95% CI 1.1%–28.0%), the patient being transported was over 450 lbs. In addition, in 2/23 cases (8.7%; 95% CI 1.1%–28.0%), the patient was uncooperative during the transport.

 There was also no association (*P* = 0.63) between location, Oklahoma City or Tulsa metropolitan service area, and cause of incident (i.e., cot failure, crew error, patient factors, surface factors). There was also no association between location and whether stretcher events occurred during patient movement (*P* = 0.11). For other analysis, events from each of the two EMSA metropolitan service areas were combined.

Since there were no patient injuries, no analysis could be performed for factors associated with patient injury. Univariate analysis of the endpoint “crew injury” revealed an association between that endpoint and the type of stretcher problem: patient factors (weight) were positively correlated with increased risk of crew injury (*P* = 0.048). Overall numbers of outcomes were too low to allow for multivariate exploration of this endpoint. No other factor was found to have a significant associated with provider injury, although low event numbers precluded a robust statistical analysis.

## 4. Discussion

In this study, the incidence of adverse events related to stretcher operations is markedly, if not surprisingly, low. There were few personnel injuries and no patient injuries during the study period. Hawthorne effect was possible, but not likely due to retrospective study nature and study time comprising one year.

 Anecdotally, the study service has rarely experienced adverse stretcher operations leading to lost work productivity and at least minor patient injury. For these reasons and in additional prevention efforts, this study was conceived to establish baseline stretcher-related adverse event and sustained injury rates to benchmark educational and operational initiative impacts. The study year was chosen at random and specifically was not chosen with any predetermination of results.

 While adverse events and resultant injury can occur during any phase of stretcher operations and due to multiple contributing factors, this study does help to delineate higher likelihood times and factors. The stretcher safety latch, mounted on the ambulance floorboard, proved prominent in this analysis of related factors. Adverse incidents more frequently occurred if the stretcher did not “catch” on this latch when being retracted from the ambulance, thereby preventing a more controlled unloading of the patient without unexpected carriage collapse, itself either partial or complete. The proper safety latch-stretcher interface, should be stressed to EMS personnel in both initial and continuing training regarding safe stretcher operations. It is important for EMS personnel to be aware of these composite results in order to further protect themselves and maintain patient safety.

 Additional investigation into this topic is greatly needed to attain firmer established insights into necessary injury-avoidance training in ambulance stretcher operations. It is discouraging to find such paucity of available peer-reviewed data addressing results of specific interventions designed to improve related safety. Perhaps a prevailing belief that little can be done outside of generic safe movement and lifting instructions persists to an extent that few academic studies of EMS stretcher operations are endeavored. Clearly, new patient transport and lifting devices designed for EMS use encourage such inquiry; our hope is this study will serve as an impetus for further evaluation of such technology.

## 5. Limitations and Future Research

An obvious limitation of the study is dependence upon EMS personnel to self-report adverse events related to stretcher operations. Within the study agency, a culture of safety is fostered, promoting self-reporting of these incidents to assist in injury prevention, limiting concern of EMS personnel regarding untoward employment repercussion for such reporting. In reality, the repercussion of “not reporting” a later discovered event is substantially more stringent. Serious patient injury in this realm would most likely be additionally discovered through patient or legal surrogate complaint, including litigation initiation. Particularly minor injuries could have been missed by EMS providers, especially if not verbalized by affected patients.

 Low absolute numbers of ambulance stretcher-related patient and EMS professional injuries occurring during the study period are markedly advantageous in injury prevention and operational efficiency paradigms. From an academic inquiry view, these low numbers are not given to form statistically robust conclusions. Thus, our results should be considered preliminary. Further similar data collection in this and other large volume EMS systems will aid in determining if a larger dataset will result in narrower confidence intervals with resultant higher statistical significance. Multivariate analysis of these suggested larger data sets will aid in determining if specific interventions are warranted against discrete types of situations more prone to ambulance stretcher-related injuries.

 Additionally, EMS systems utilizing different stretchers may not find direct applicability of these findings.

## 6. Conclusions

In this large, urban EMS system, the incidence of injury related to stretcher operations in the one-year study period is markedly low, with few personnel injuries and no patient injuries incurred. EMS personnel should be aware of the risk of injury to themselves that can occur during stretcher operations when moving morbidly obese and/or combative patients. Safety for EMS personnel and patients could be advanced by educational initiatives that highlight specific events and conditions contributing to stretcher-related adverse events.

## Figures and Tables

**Table 1:** Timing of stretcher-related adverse events (*n* = 23).

Timing of event	Number of events	Percent of total events	95% CI
Unloading	15	65.2	42.7–83.6
Loading	5	21.7	7.5–43.7
Surface Movement	3	13.0	2.8–33.6

**Table 2:** Contributing factors to stretcher-related adverse events (*n* = 23).

Contributing factor	Number of events	Percent of total events	95% CI
Latch malfunction	14	60.9	38.5–80.3
Surface condition	4	17.4	5.0–38.8
Equipment failure	3	13.0	2.8–33.6
Pt weight >450 lbs	2	8.7	1.1–28.0
Pt uncooperative	2	8.7	1.1–28.0

## References

[1] Wang HE, Weaver MD, Abo BN, Kaliappan R, Fairbanks RJ (2009). Ambulance stretcher adverse events. *Quality and Safety in Health Care*.

[2] Aasa U, Ängquist K-A, Barnekow-Bergkvist M (2008). The effects of a 1-year physical exercise programme on development of fatigue during a simulated ambulance work task. *Ergonomics*.

[3] Leyk D, Rohde U, Erley O (2007). Maximal manual stretcher carriage: performance and recovery of male and female ambulance workers. *Ergonomics*.

[4] Cooper G, Ghassemieh E (2007). Risk assessment of patient handling with ambulance stretcher systems (ramp/(winch), easi-loader, tail-lift) using biomechanical failure criteria. *Medical Engineering & Physics*.

[5] Barnekow-Bergkvist M, Aasa U, Ängquist K-A, Johansson H (2004). Prediction of development of fatigue during a simulated ambulance work task from physical performance tests. *Ergonomics*.

[6] U. S. Census Bureau American FactFinder fact sheet: State of Oklahoma. http://factfinder2.census.gov/faces/tableservices/jsf/pages/productview.xhtml?pid=DEC_10_PL_GCTPL2.ST13&prodType=table.

[7] Federal Specifications for Ambulances

